# Supplementary material for: Hemiacetalmeroterpenoids A–C and Astellolide Q with Antimicrobial Activity from the Marine-Derived Fungus Penicillium sp. N-5
Source: Mar Drugs. 2022 Aug 13;20(8):514. doi: 10.3390/md20080514 (PMC9410149; doi:10.3390/md20080514)
Supplement: Supplementary file 1 [file marinedrugs-20-00514-s001.zip › marinedrugs-1871289-supplementary.pdf]

Hemiacetalmeroterpenoids A-C and Astellolide Q with Antimicrobial Activity from the Marine-Derived Fungus *Penicillium* sp. N-5

Tao Chen, Wencong Yang, Taobo Li, Yihao Yin, Yufeng Liu, Bo Wang *, Zhigang She *

School of Chemistry, Sun Yat-Sen University, Guangzhou 510275, China

* Correspondence: ceswb@mail.sysu.edu.cn (B.W.); cesshzhg@mail.sysu.edu.cn (Z.S.)

Supporting information

Figure S1. HRESIMS spectrum of compound **1**........3
Figure S2. ^{1}H NMR spectrum of compound **1** (600 MHz, CD_3OD)........3
Figure S3. ^{13}C NMR spectrum of compound **1** (150 MHz, CD_3OD)........4
Figure S4. DEPT135 spectrum of compound **1** (150 MHz, CD_3OD)........4
Figure S5. HSQC spectrum of compound **1** (CD_3OD)........5
Figure S6. H, H-COSY spectrum of compound **1** (CD_3OD)........5
Figure S7. HMBC spectrum of compound **1** (CD_3OD)........6
Figure S8. NOE spectrum of compound **1** (CD_3OD)........6
Figure S9. HRESIMS spectrum of compound **2**........7
Figure S10. ^{1}H NMR spectrum of compound **2** (600 MHz, CD_3OD)........7
Figure S11. ^{13}C NMR spectrum of compound **2** (150 MHz, CD_3OD)........8
Figure S12. DEPT135 spectrum of compound **2** (150 MHz, CD_3OD)........8
Figure S13. HSQC spectrum of compound **2** (CD_3OD)........9
Figure S14. H, H-COSY spectrum of compound **2** (CD_3OD)........9
Figure S15. HMBC spectrum of compound **2** (CD_3OD)........10
Figure S17. HRESIMS spectrum of compound **3**........11
Figure S18. ^{1}H NMR spectrum of compound **3** (600 MHz, CD_3OD)........11
Figure S19. ^{13}C NMR spectrum of compound **3** (150 MHz, CD_3OD)........12
Figure S20. HSQC spectrum of compound **3** (CD_3OD)........12
Figure S21. H, H-COSY spectrum of compound **3** (CD_3OD)........13
Figure S22. HMBC spectrum of compound **3** (CD_3OD)........13
Figure S23. NOE spectrum of compound **3** (CD_3OD)........14
Figure S24. NOE spectrum of compound **14** (CD_3OD)........14
Figure S25. HRESIMS spectrum of compound **15**........15
Figure S26. ^{1}H NMR spectrum of compound **15** (400 MHz, CD_3OD)........15
Figure S27. ^{13}C NMR spectrum of compound **15** (100 MHz, CD_3OD)........16
Figure S28. DEPT135 spectrum of compound **15** (150 MHz, CD_3OD)........16
Figure S29. HSQC spectrum of compound **15** (CD_3OD)........17
Figure S30. H, H-COSY spectrum of compound **15** (CD_3OD)........17
Figure S31. HMBC spectrum of compound **15** (CD_3OD)........18
Figure S32. NOE spectrum of compound **15** (CD_3OD)........18
Figure S33. UV and ECD of compound **1** (CD_3OD)........19
Figure S34. UV and ECD of compound **2** (CD_3OD)........19
Figure S35. UV and ECD of compound **3** (CD_3OD)........19
Figure S36. UV and ECD of compound **14** (CD_3OD)........20
Figure S37. UV and ECD of compound **15** (CD_3OD)........20

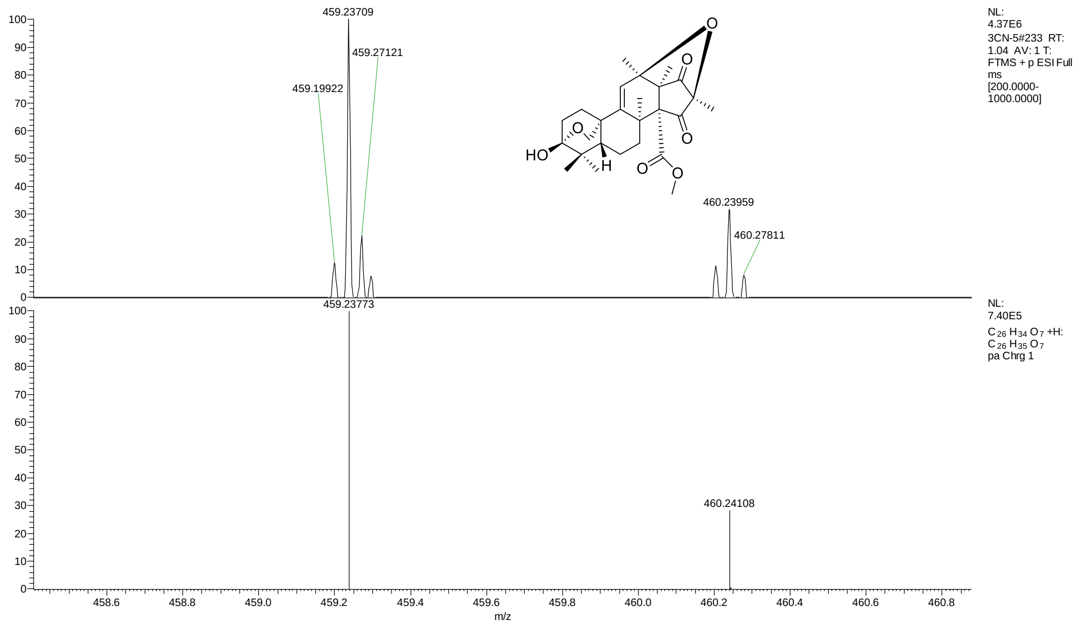

Figure S1. HRESIMS spectrum of compound **1**

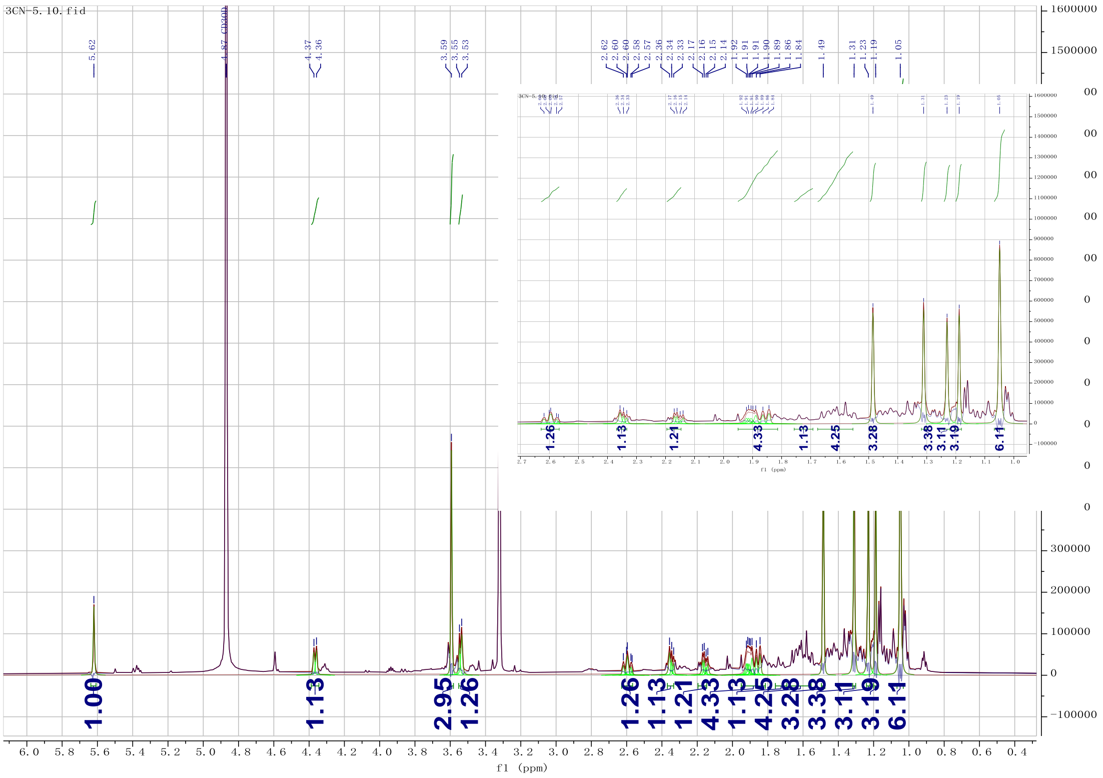

Figure S2. ^{1}H NMR spectrum of compound **1** (600 MHz, CD_3OD)

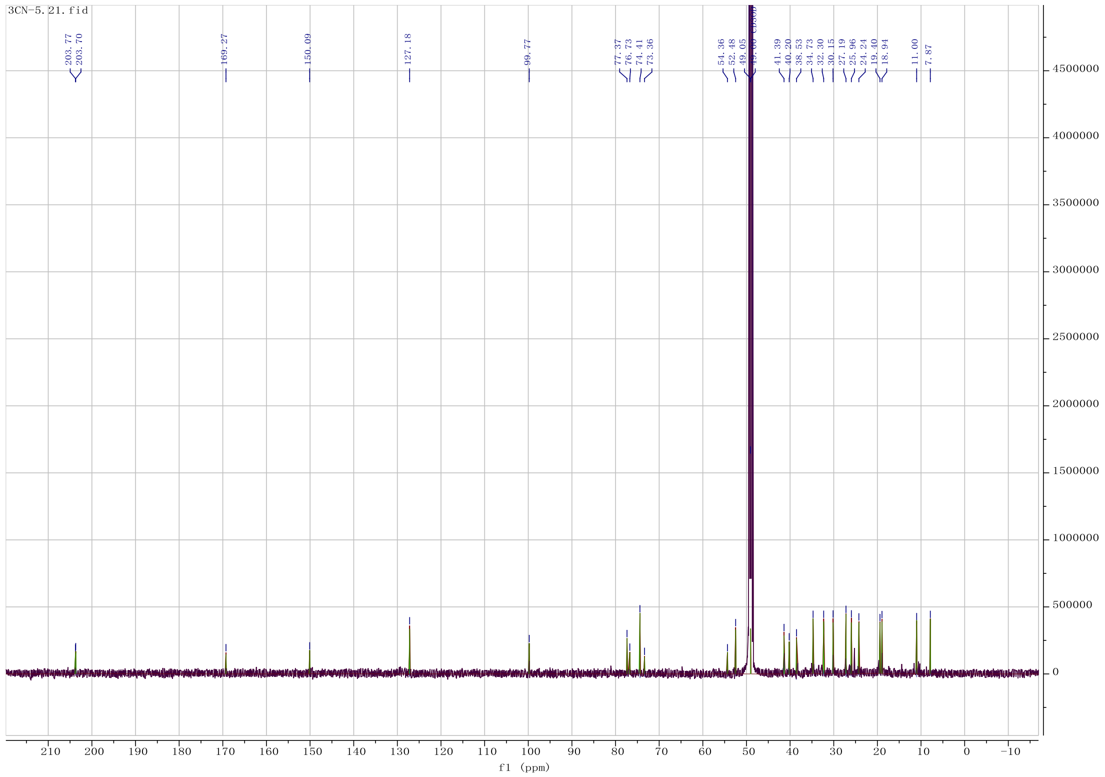

Figure S3. ^{13}C NMR spectrum of compound **1** (150 MHz, CD_3OD)

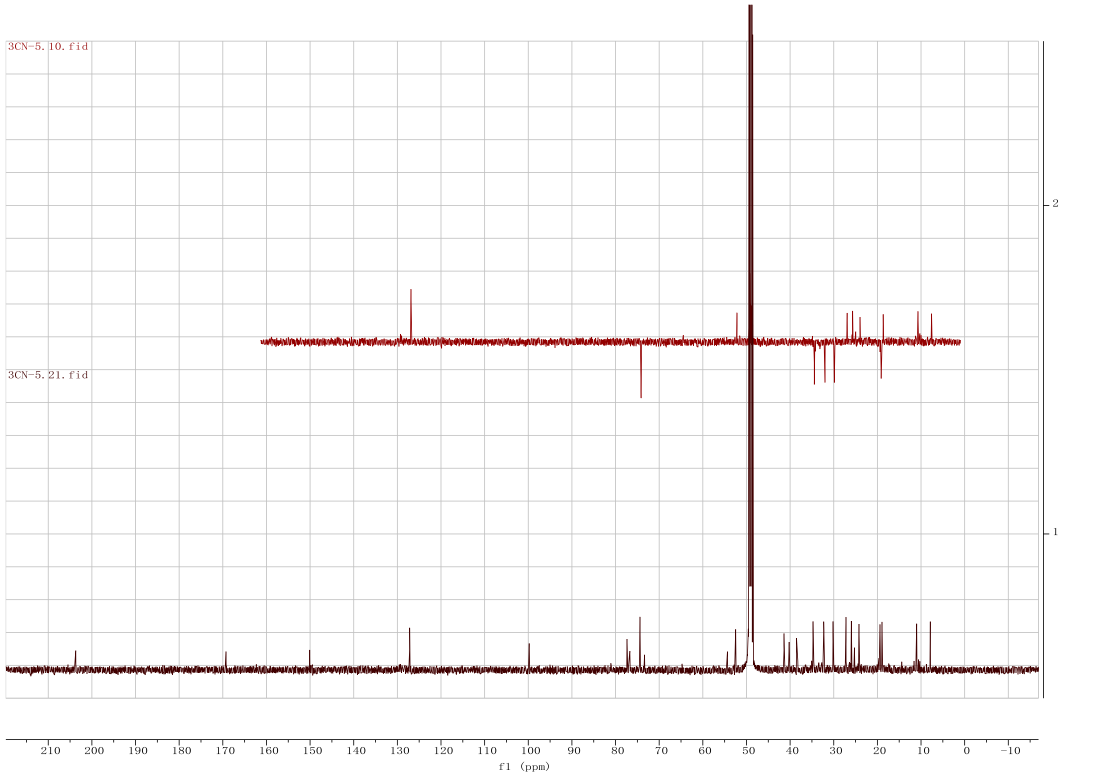

Figure S4. DEPT135 spectrum of compound **1** (150 MHz, CD_3OD)

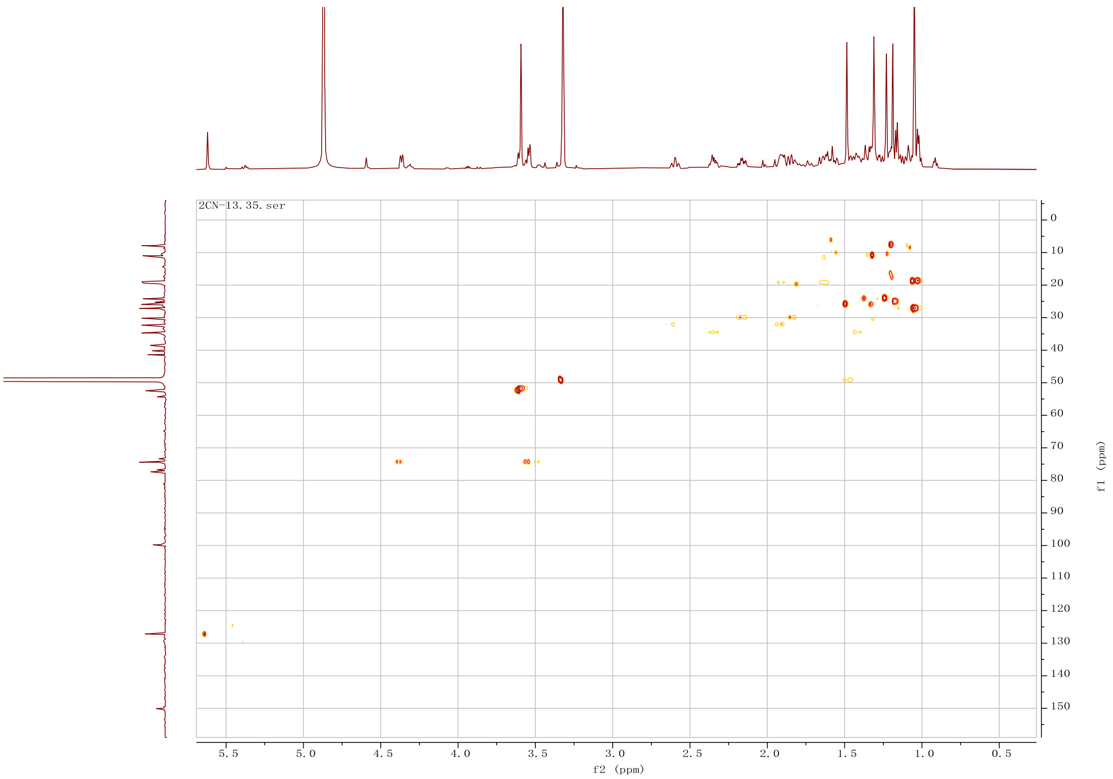

Figure S5. HSQC spectrum of compound **1** (CD_3OD)

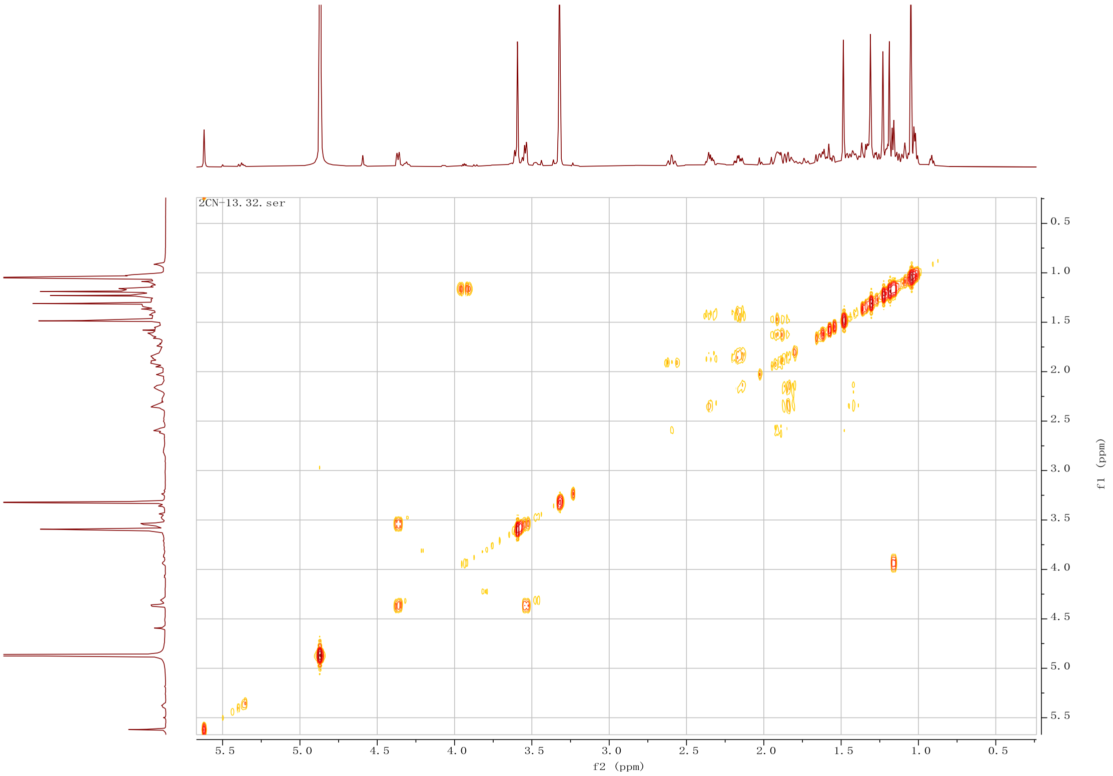

Figure S6. H, H-COSY spectrum of compound **1** (CD_3OD)

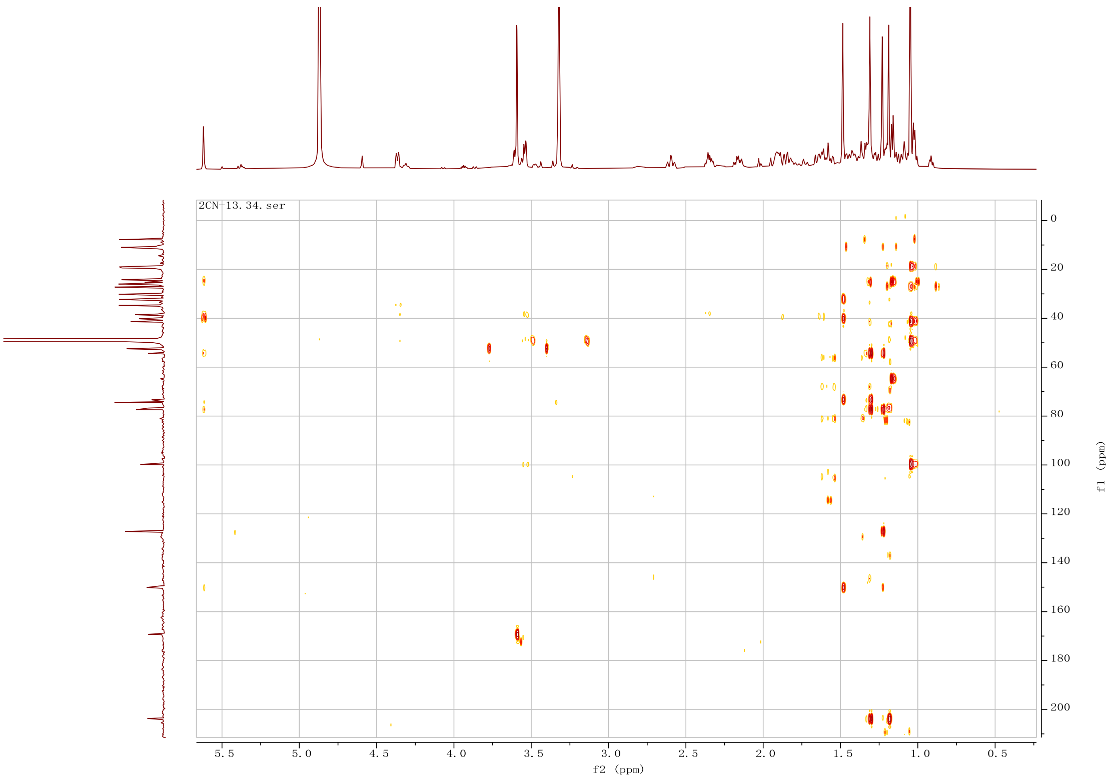

Figure S7. HMBC spectrum of compound **1** (CD_3OD)

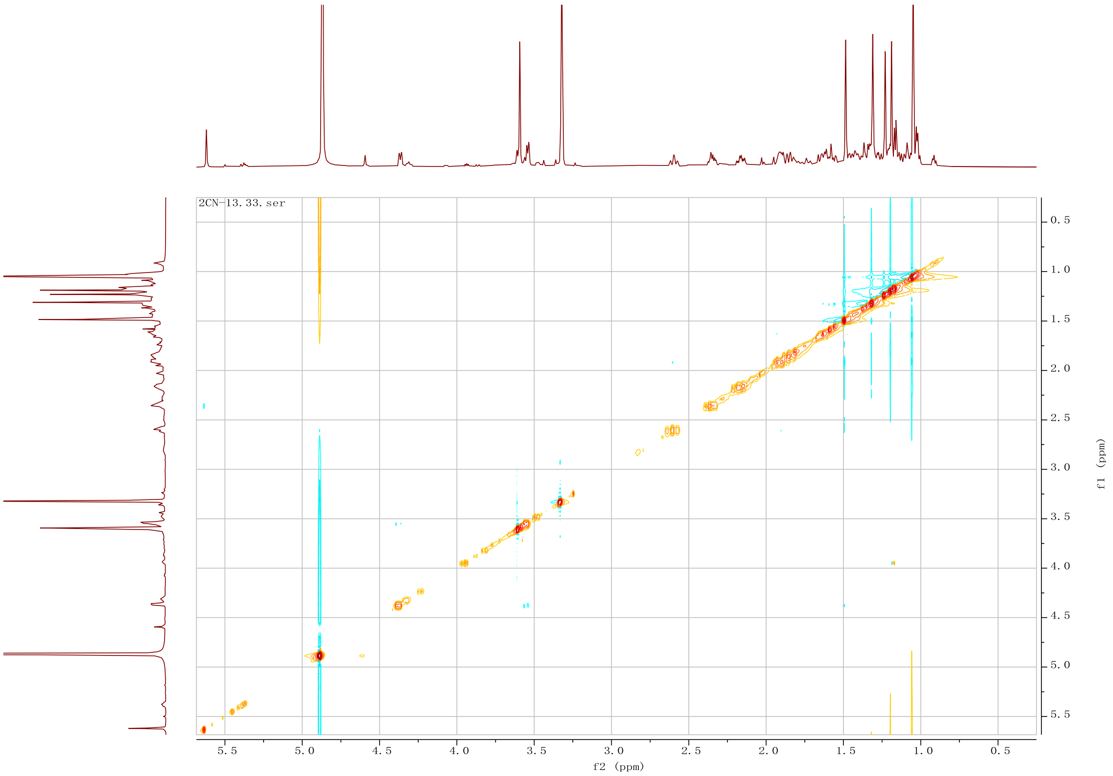

Figure S8. NOE spectrum of compound **1** (CD_3OD)

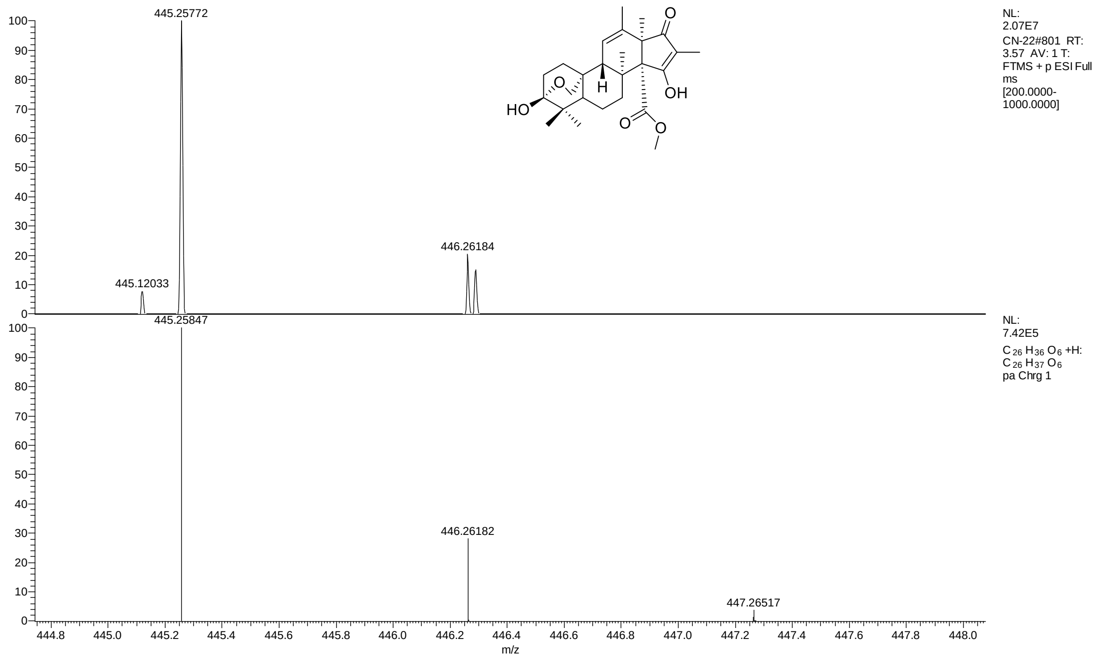

Figure S9. HRESIMS spectrum of compound **2**

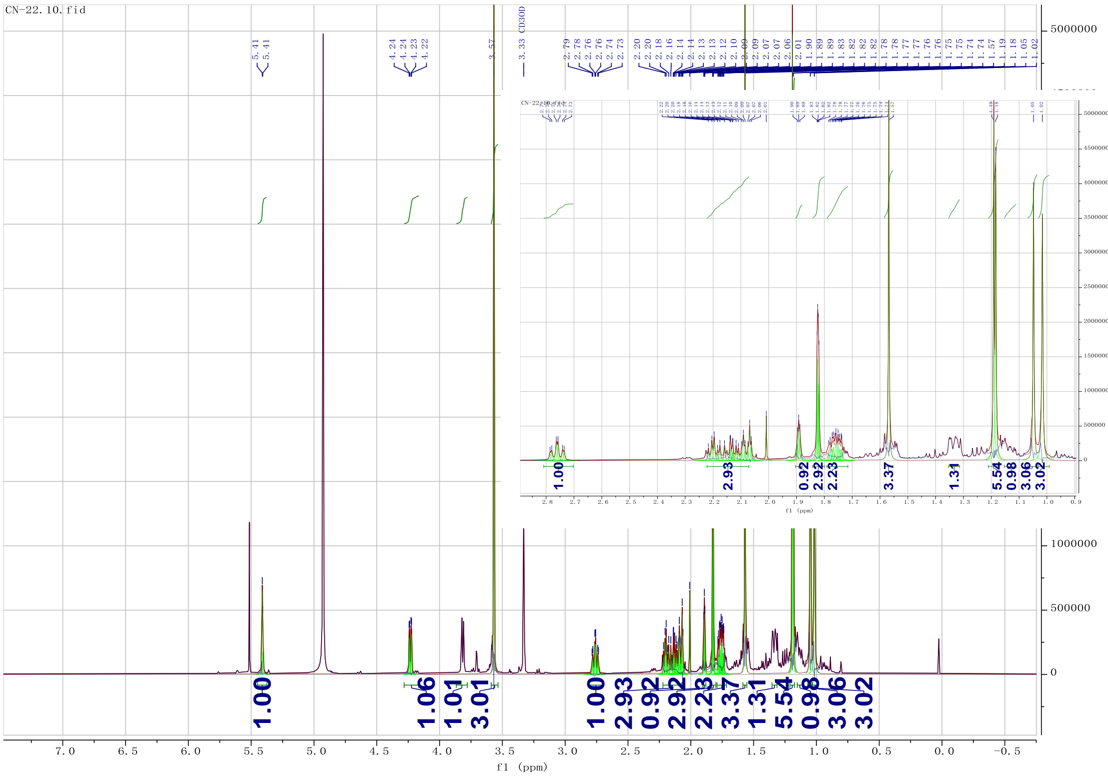

Figure S10. ^{1}H NMR spectrum of compound **2** (600 MHz, CD_3OD)

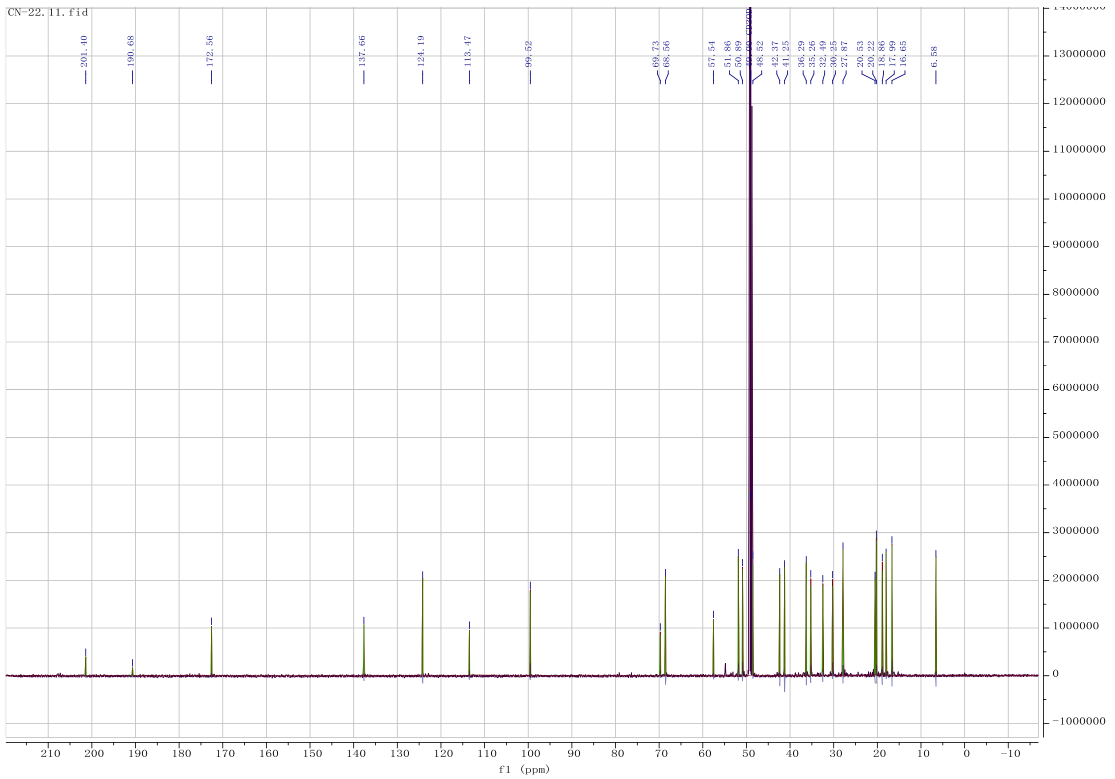

Figure S11. ^{13}C NMR spectrum of compound **2** (150 MHz, CD_3OD)

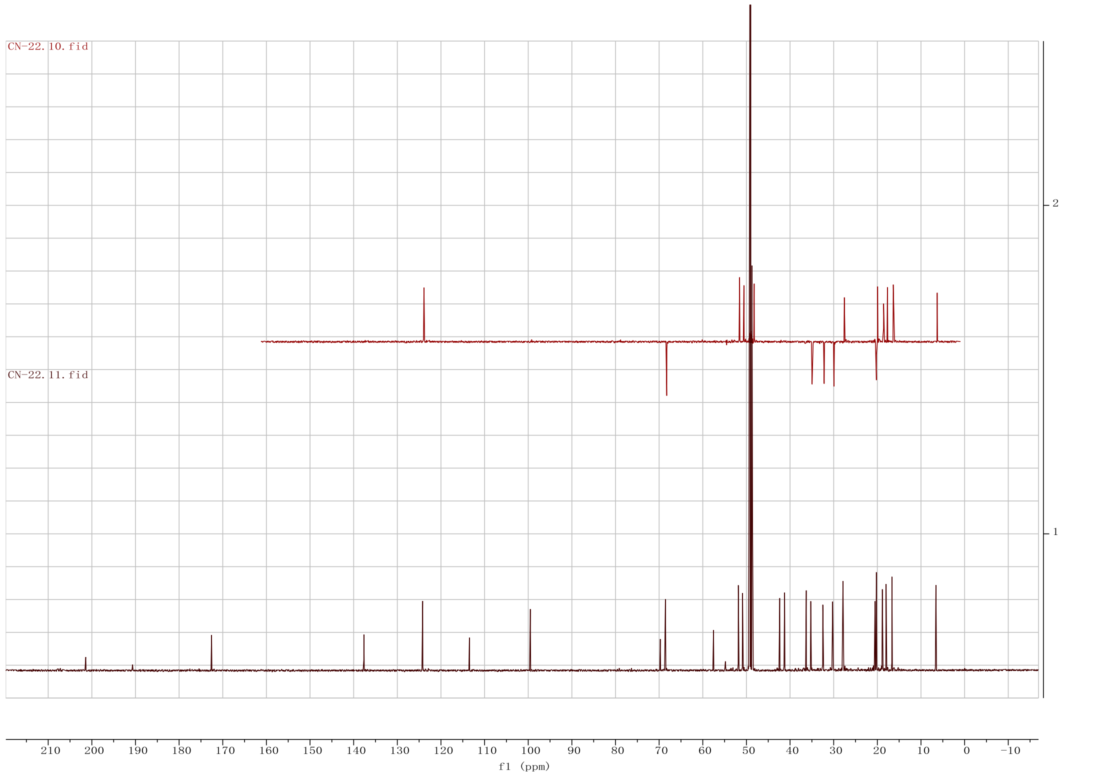

Figure S12. DEPT135 spectrum of compound **2** (150 MHz, CD_3OD)

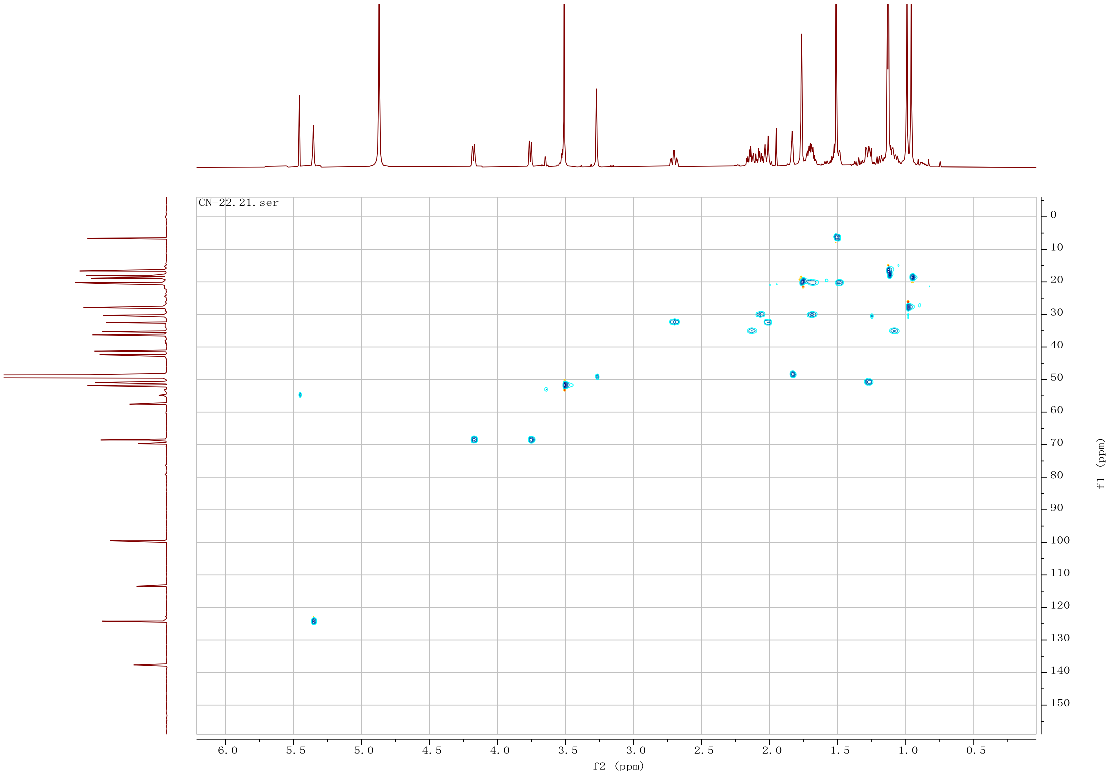

Figure S13. HSQC spectrum of compound **2** (CD_3OD)

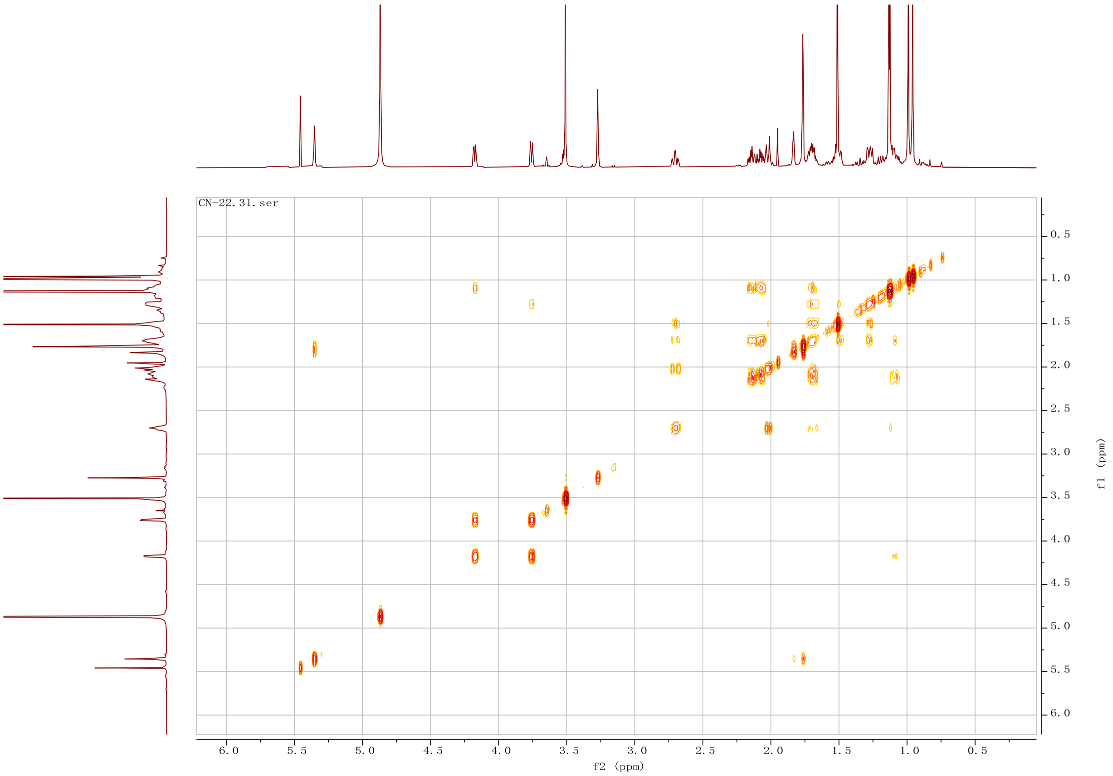

Figure S14. H, H-COSY spectrum of compound **2** (CD_3OD)

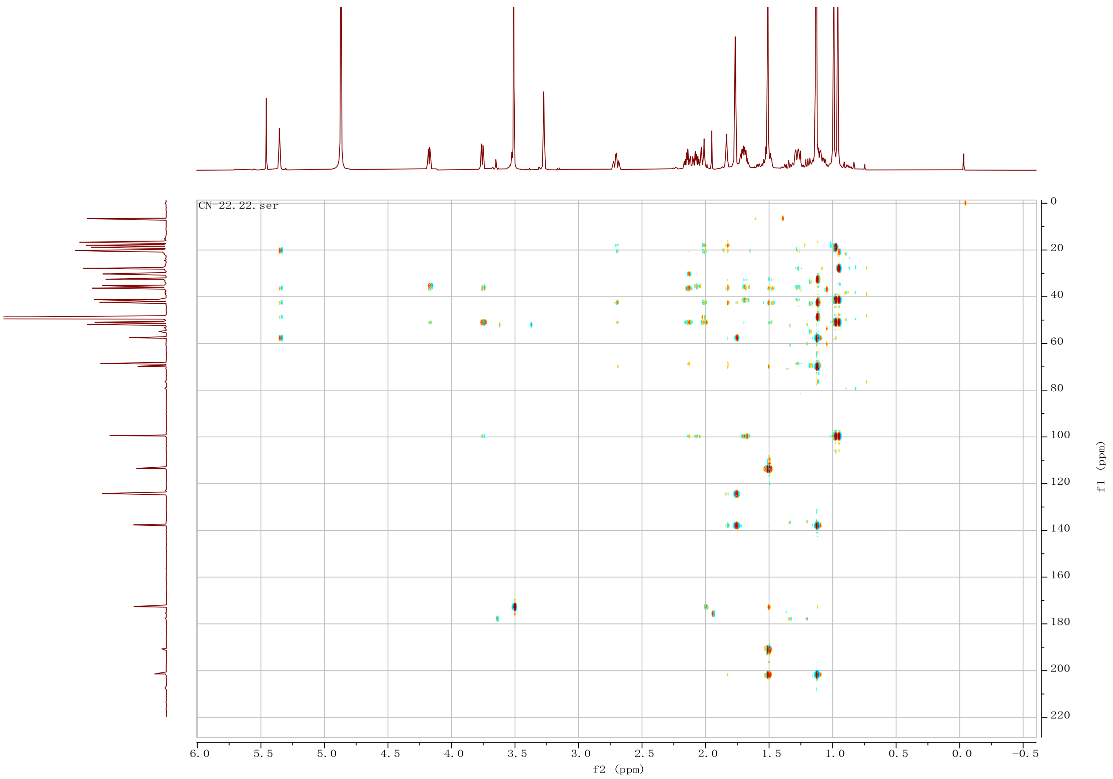

Figure S15. HMBC spectrum of compound **2** (CD_3OD)

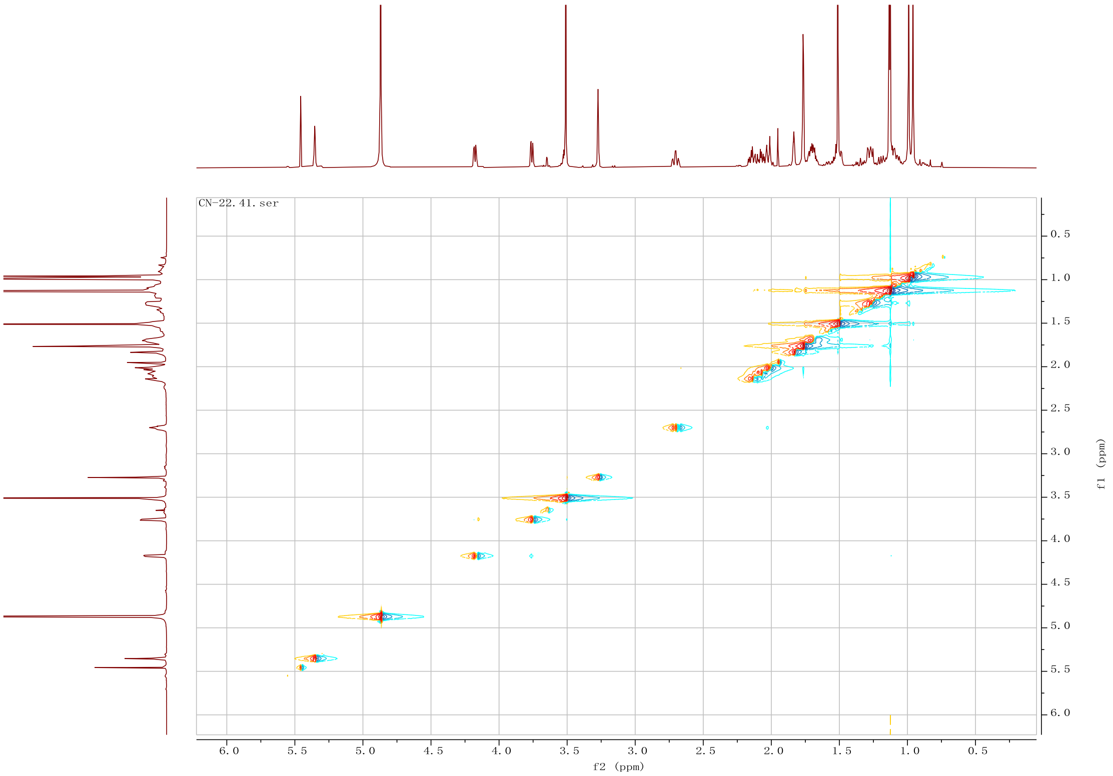

Figure S16. NOE spectrum of compound 2 (CD3OD)

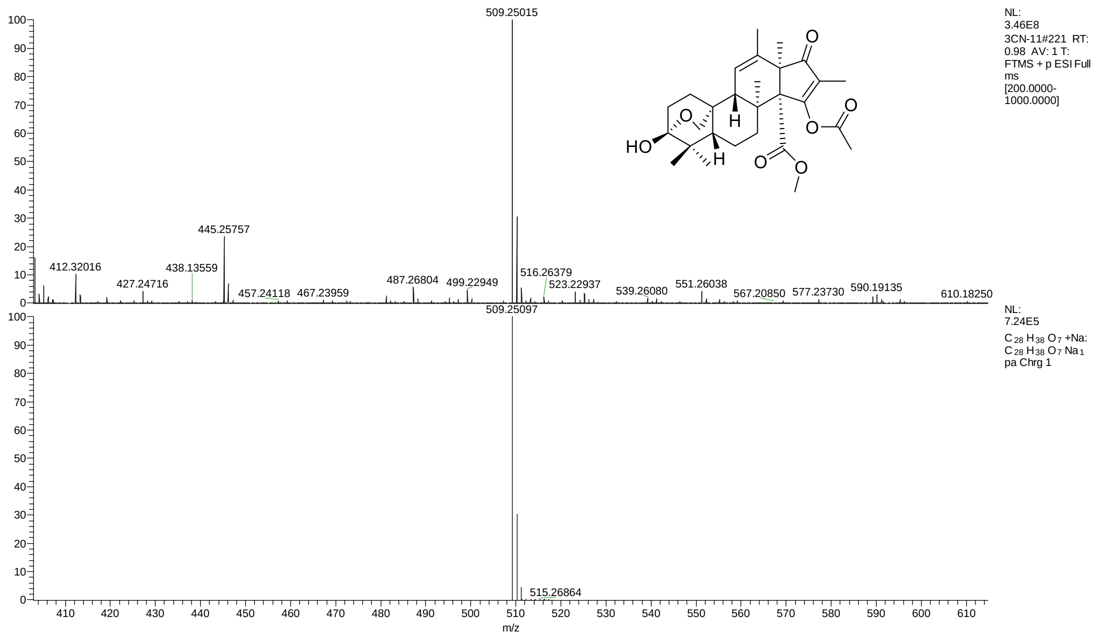

Figure S17. HRESIMS spectrum of compound **3**

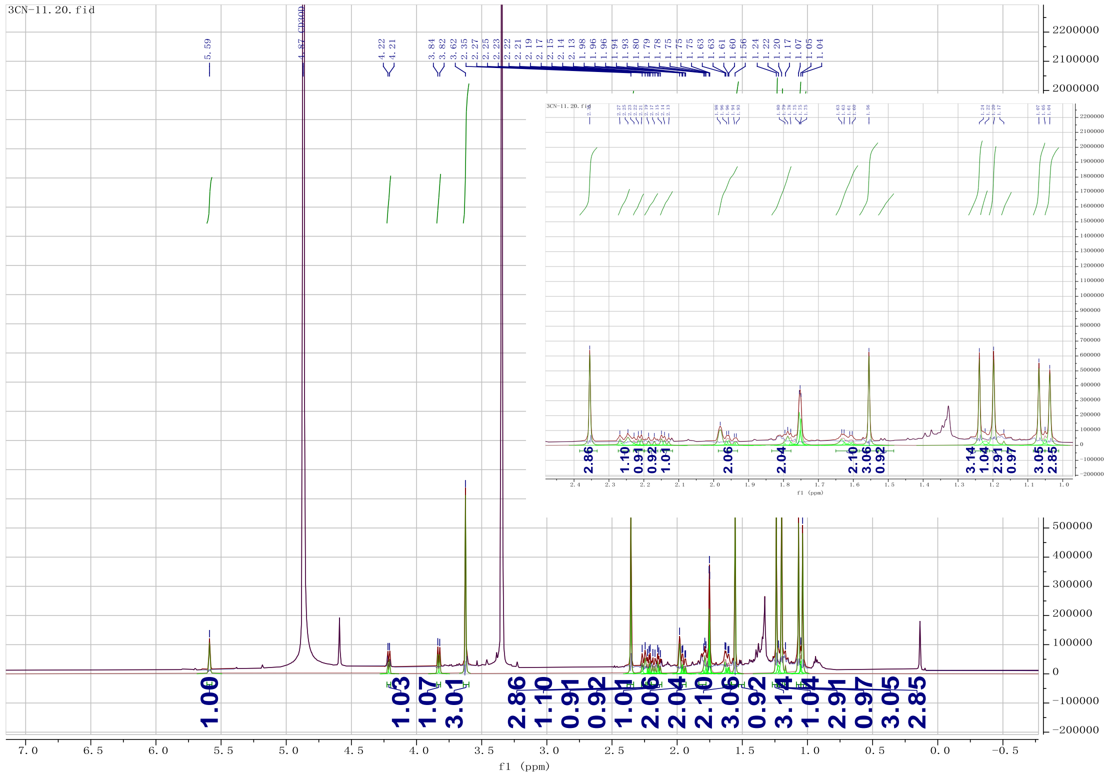

Figure S18. ^{1}H NMR spectrum of compound **3** (600 MHz, CD_3OD)

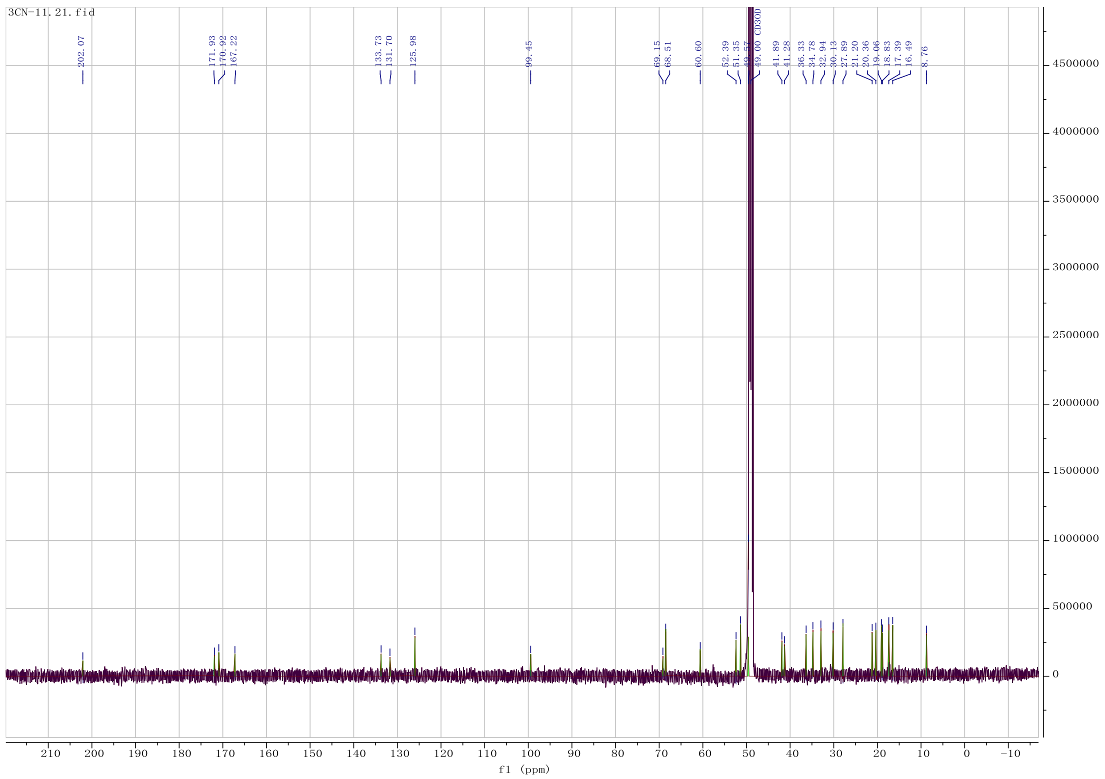

Figure S19. ^{13}C NMR spectrum of compound **3** (150 MHz, CD_3OD)

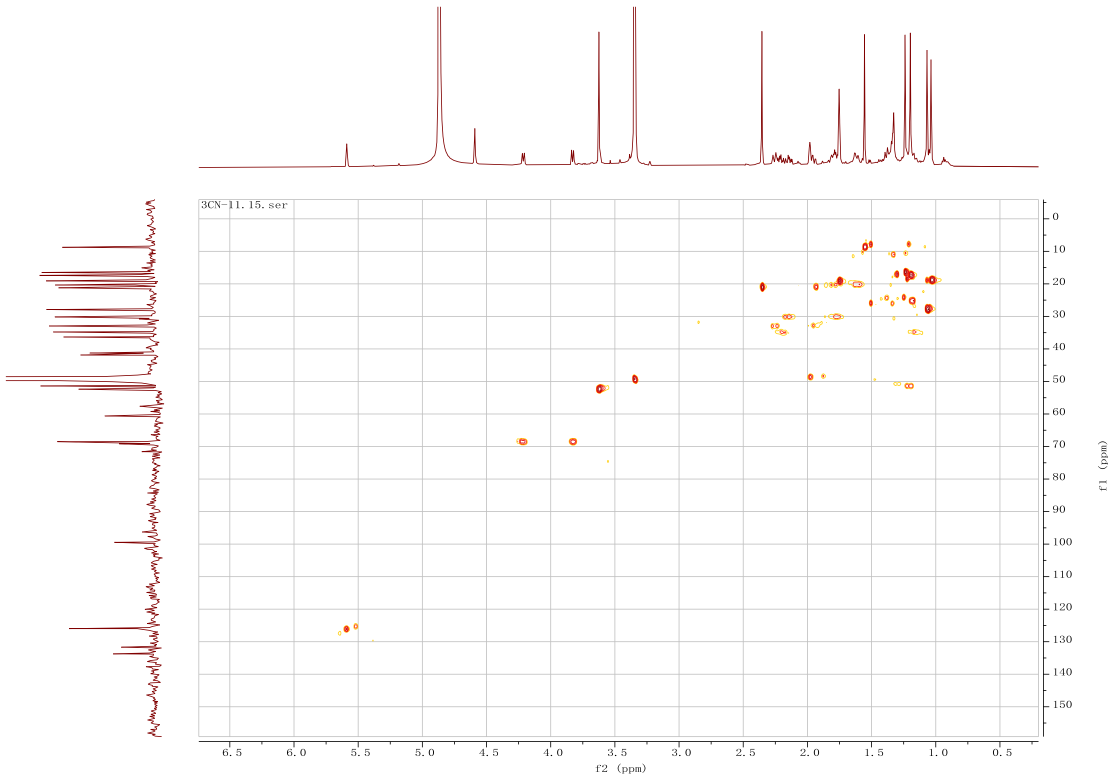

Figure S20. HSQC spectrum of compound **3** (CD_3OD)

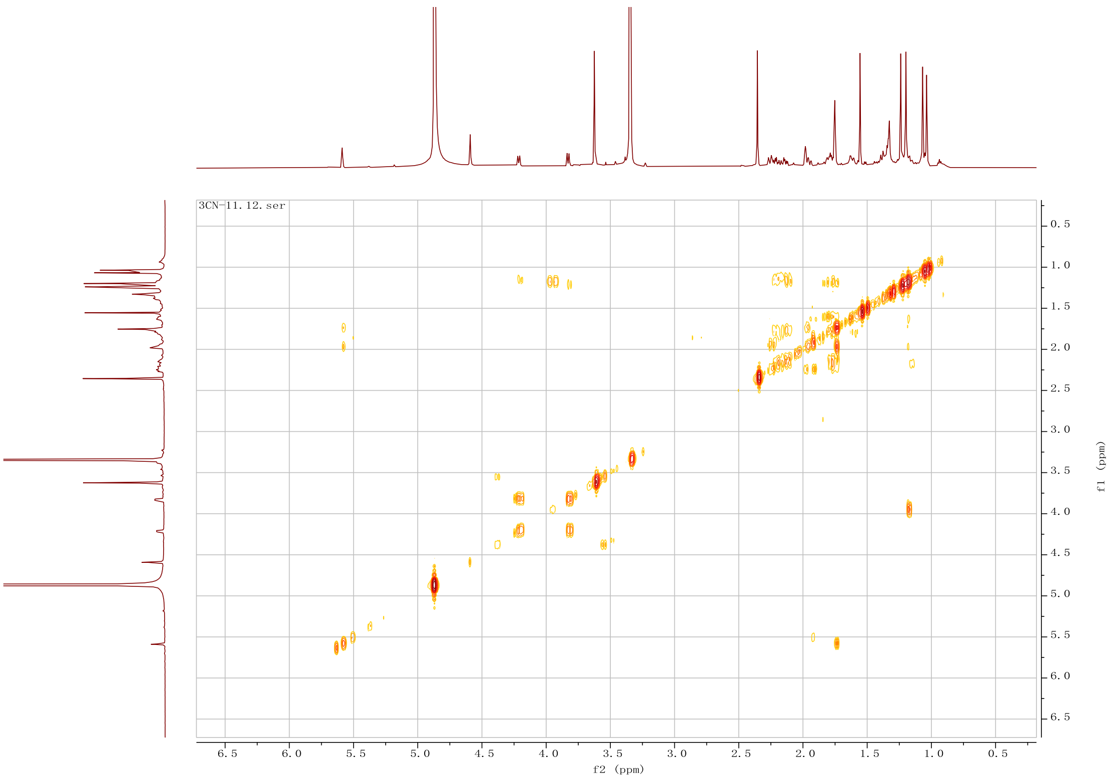

Figure S21. H, H-COSY spectrum of compound **3** (CD_3OD)

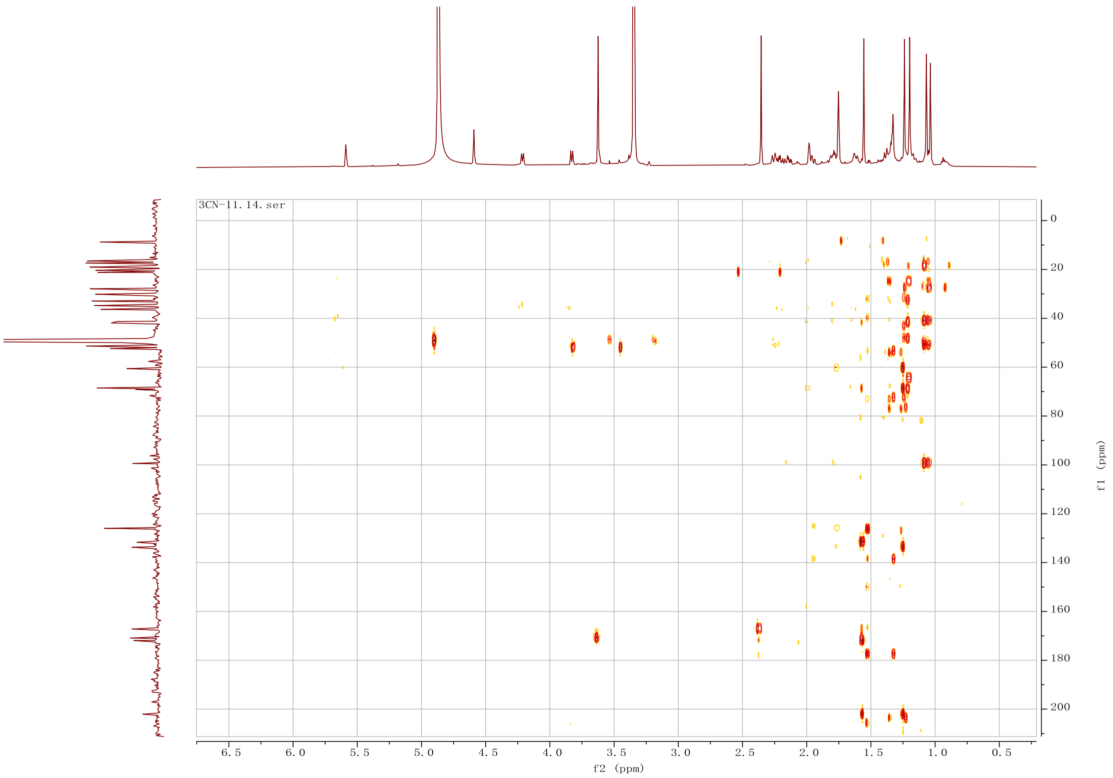

Figure S22. HMBC spectrum of compound **3** (CD_3OD)

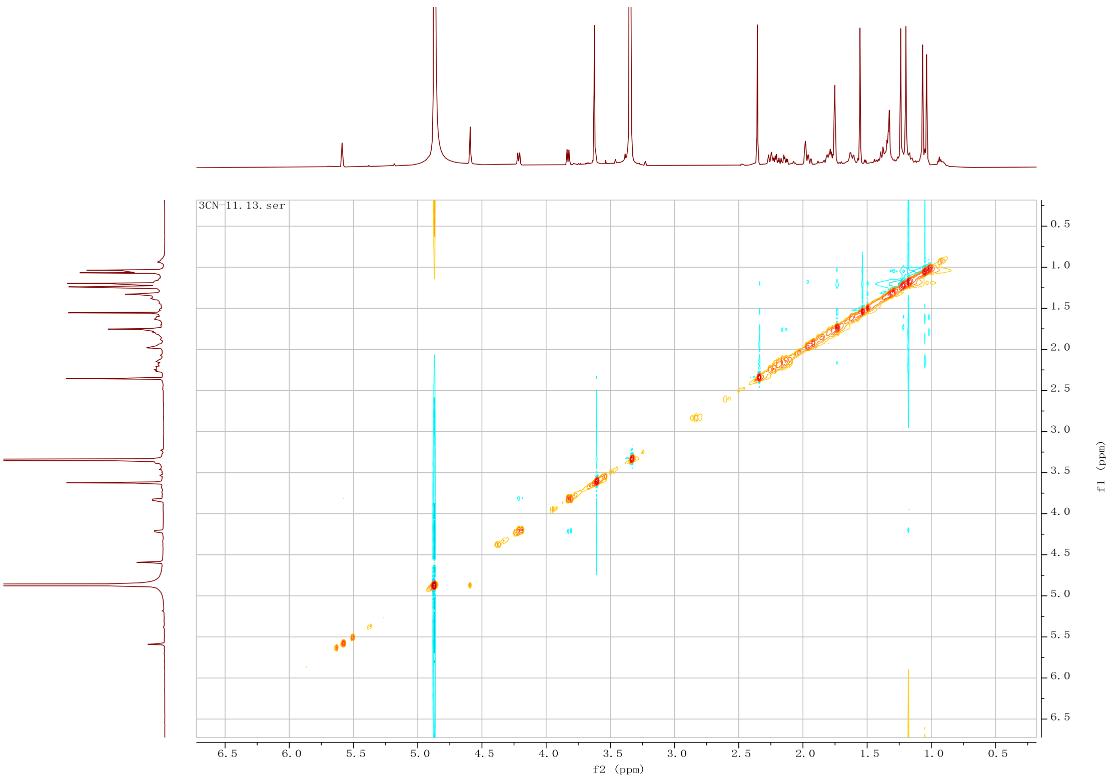

Figure S23. NOE spectrum of compound **3** (CD_3OD)

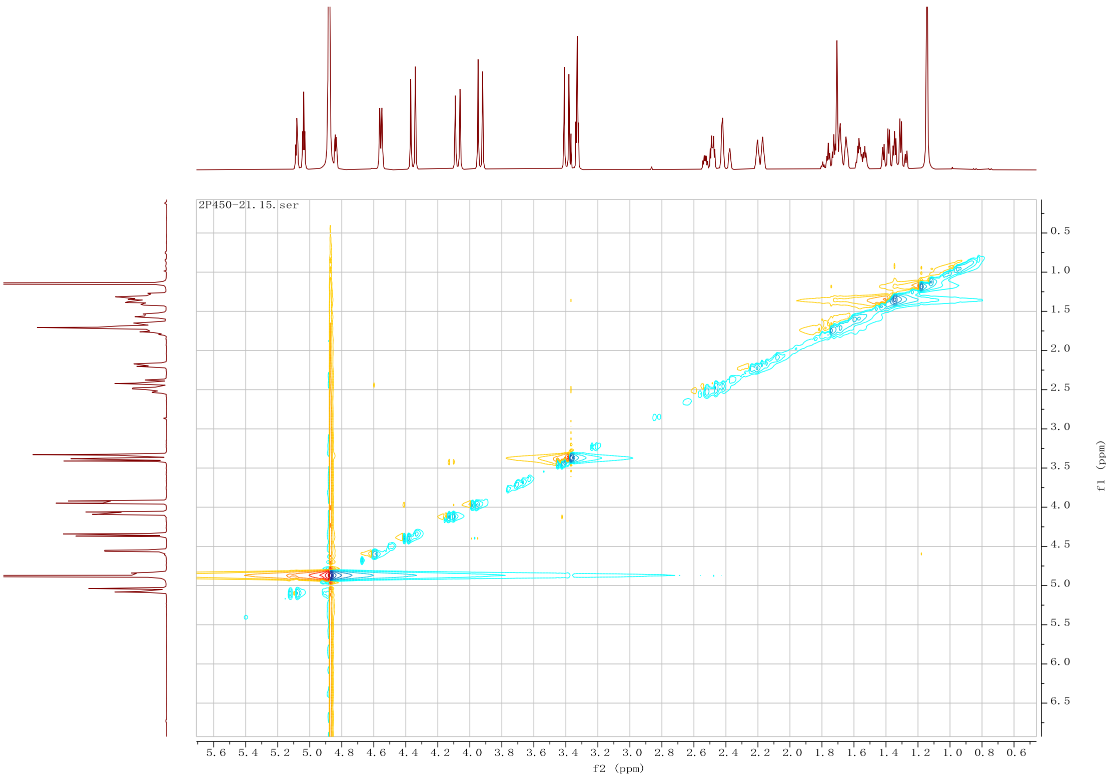

Figure S24. NOE spectrum of compound **14** (CD_3OD)

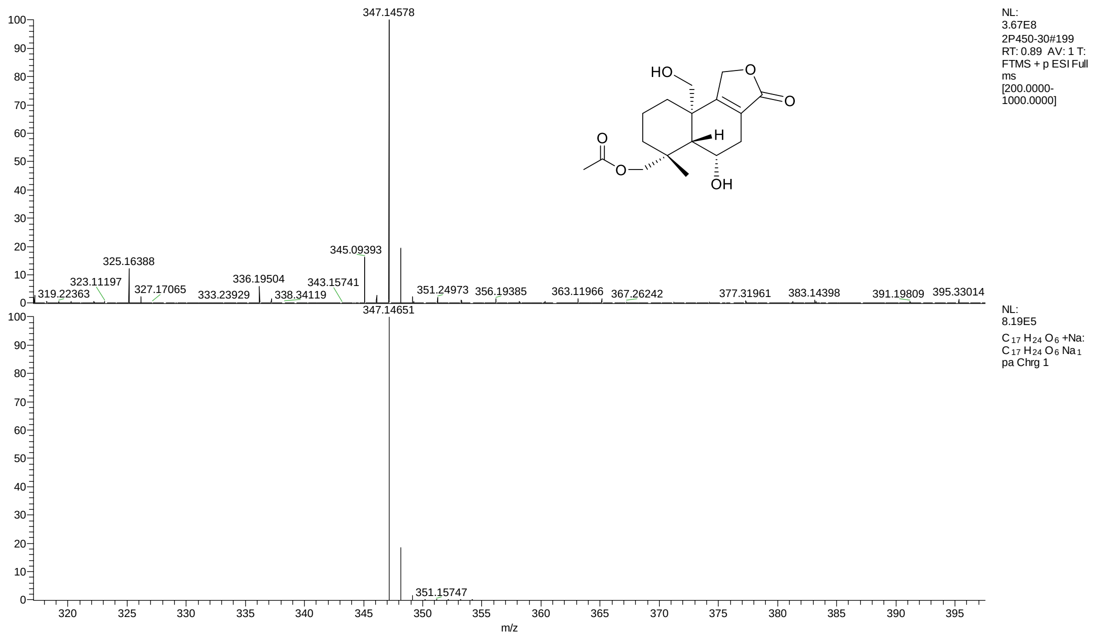

Figure S25. HRESIMS spectrum of compound **15**

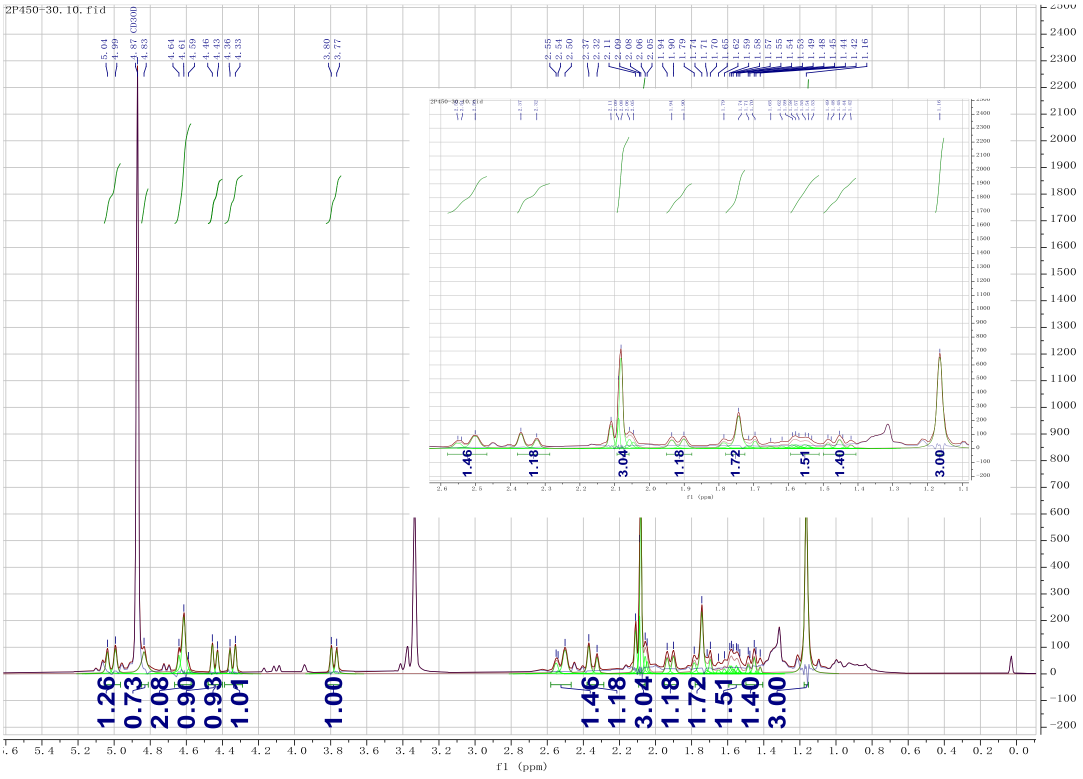

Figure S26. ^{1}H NMR spectrum of compound **15** (400 MHz, CD_3OD)

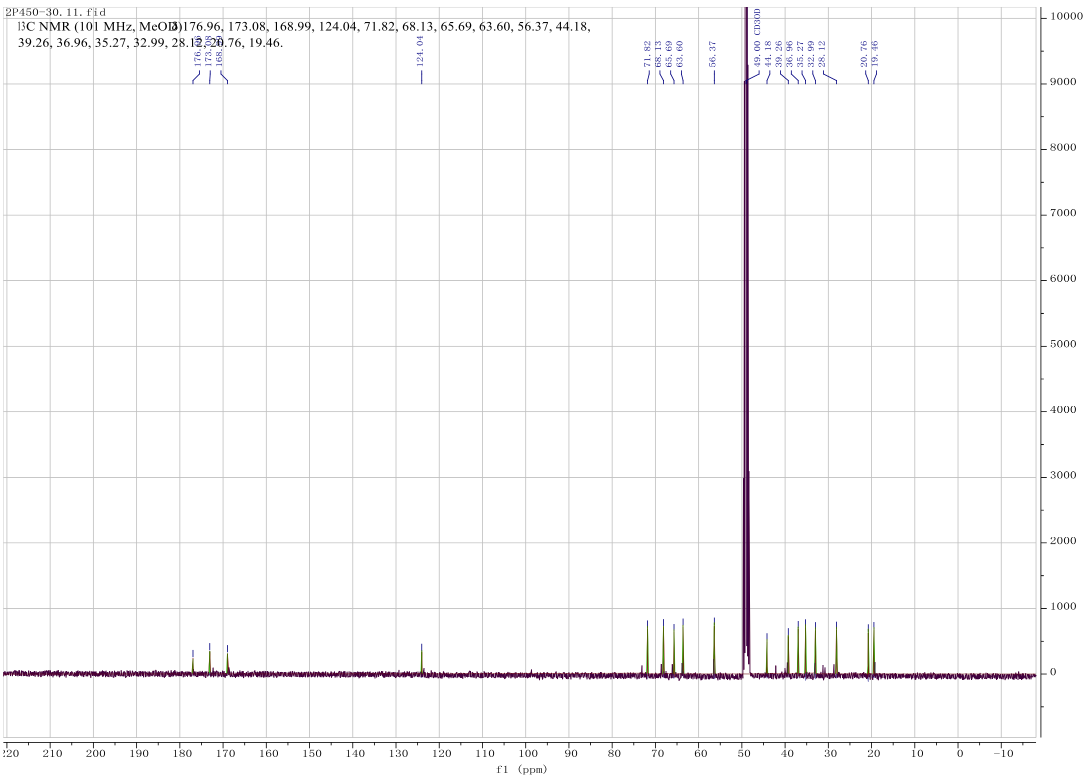

Figure S27. ^{13}C NMR spectrum of compound **15** (100 MHz, CD_3OD)

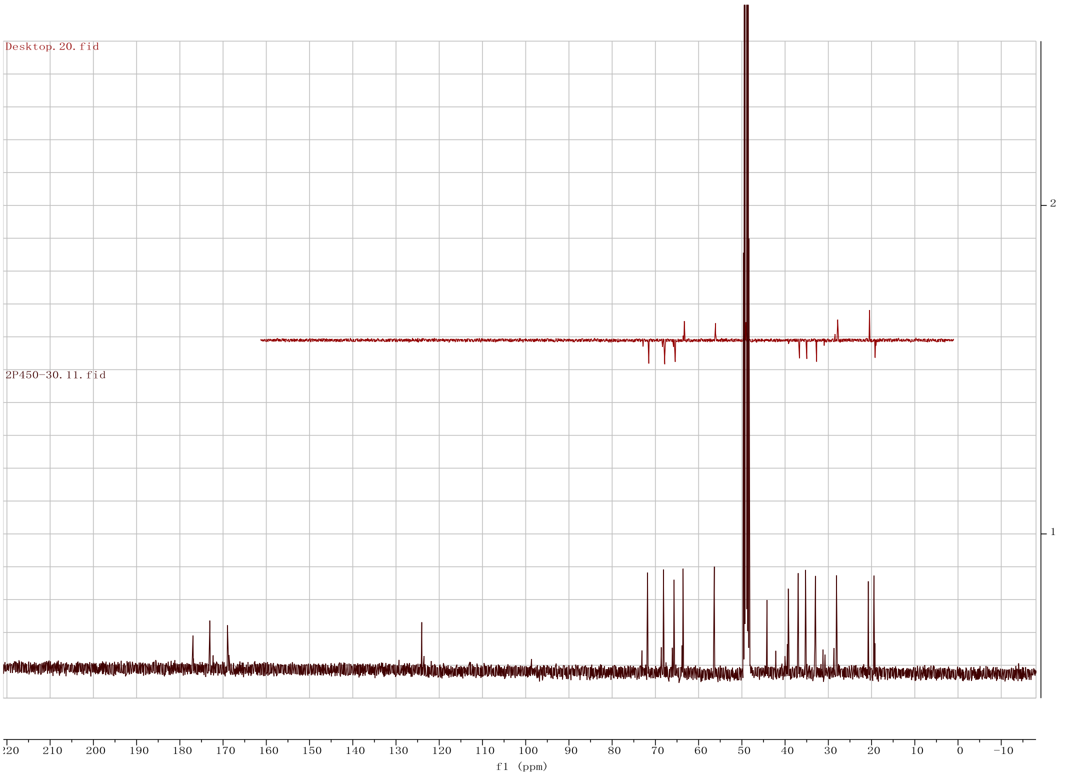

Figure S28. DEPT135 spectrum of compound **15** (150 MHz, CD_3OD)

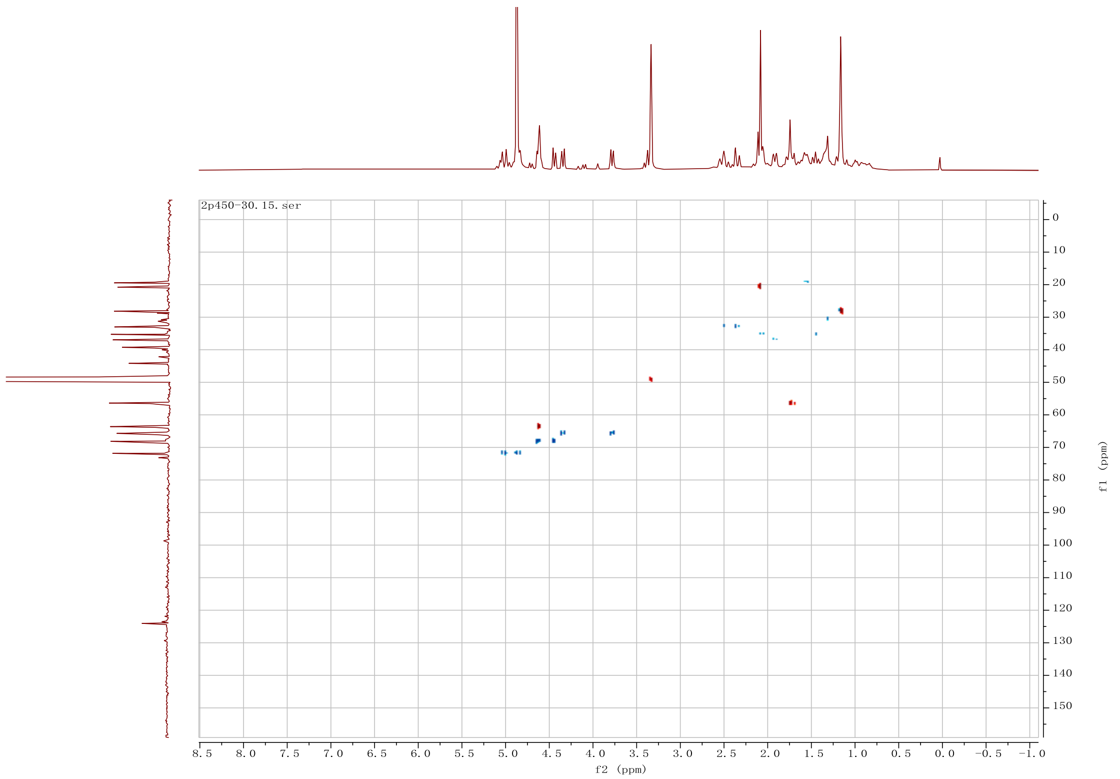

Figure S29. HSQC spectrum of compound **15** (CD_3OD)

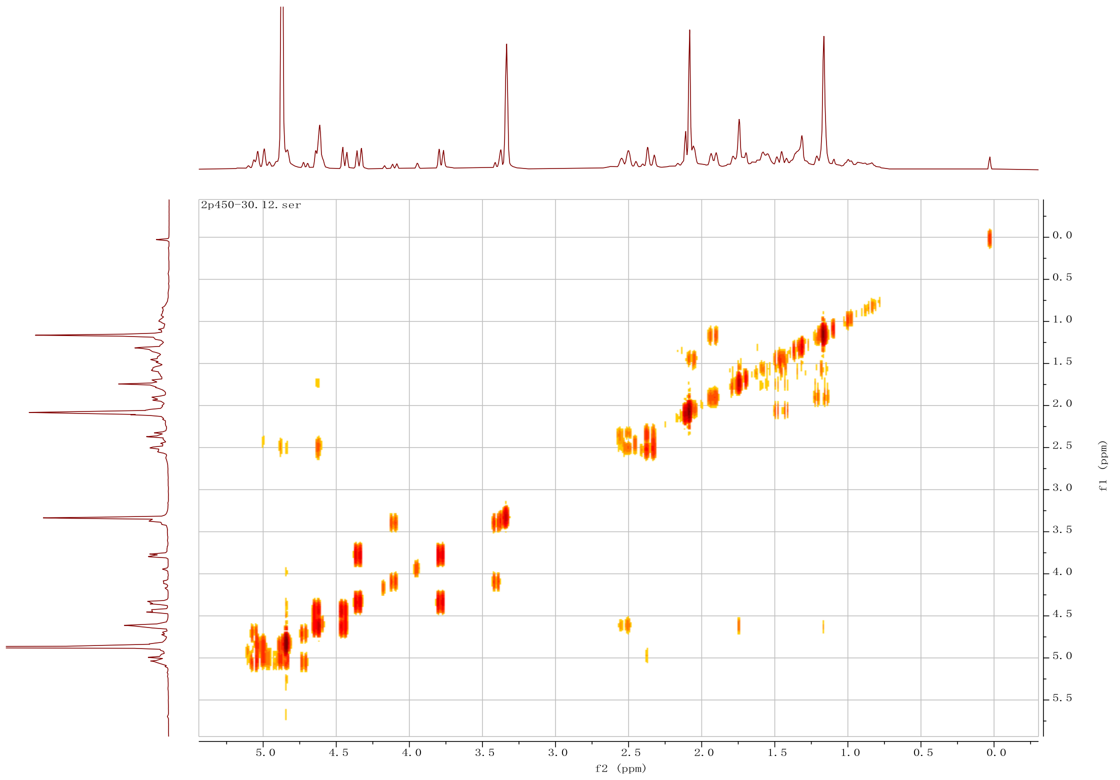

Figure S30. H, H-COSY spectrum of compound **15** (CD_3OD)

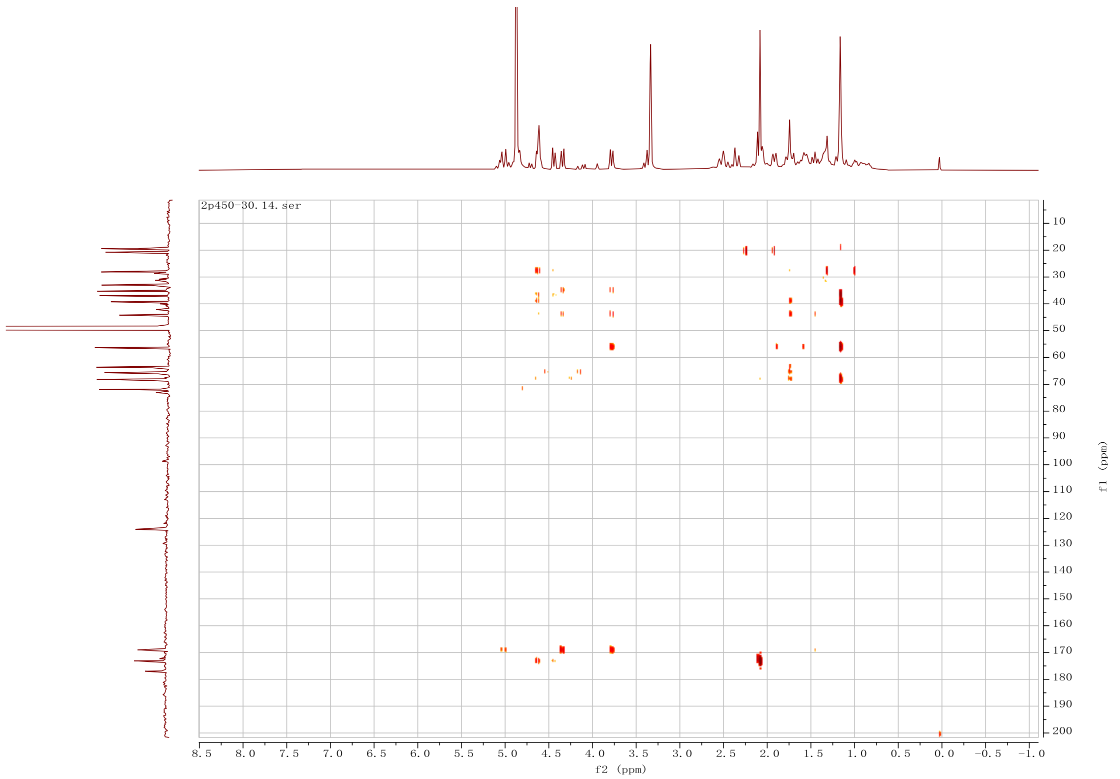

Figure S31. HMBC spectrum of compound **15** (CD_3OD)

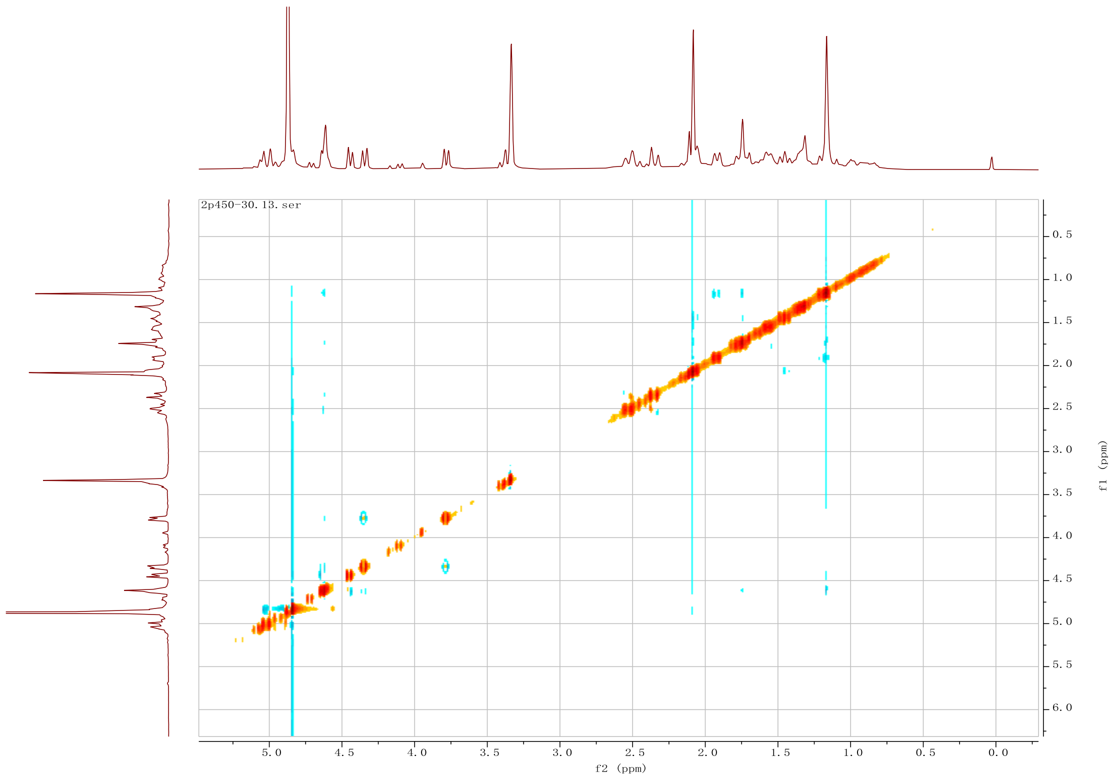

Figure S32. NOE spectrum of compound **15** (CD_3OD)

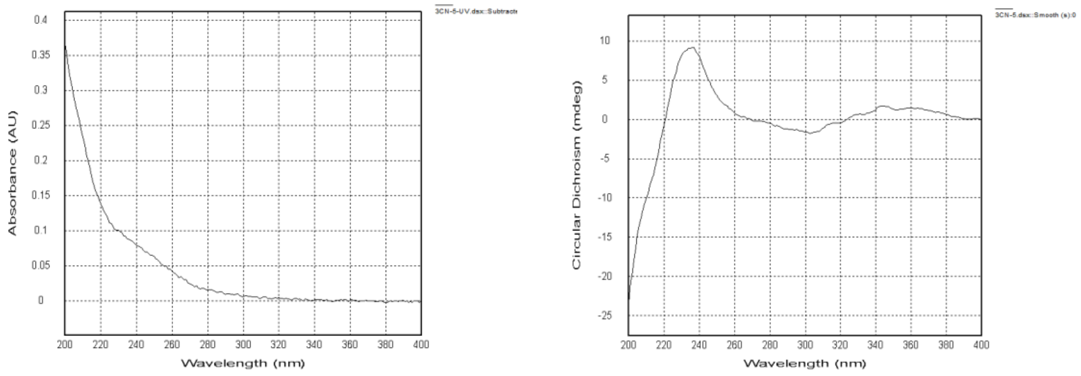

Figure S33. UV and ECD of compound **1** (CD_3OD)

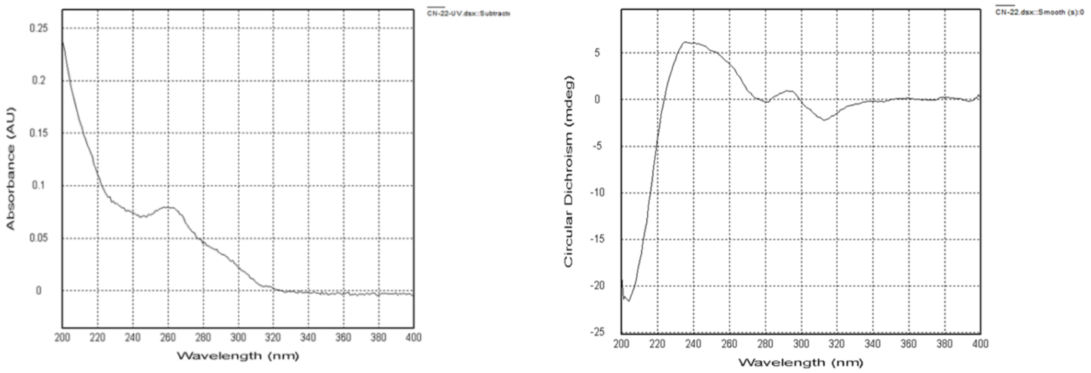

Figure S34. UV and ECD of compound **2** (CD_3OD)

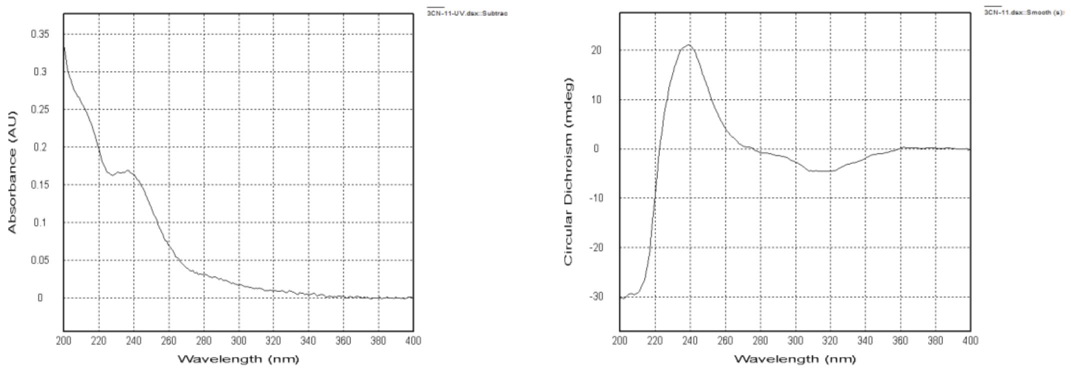

Figure S35. UV and ECD of compound **3** (CD_3OD)

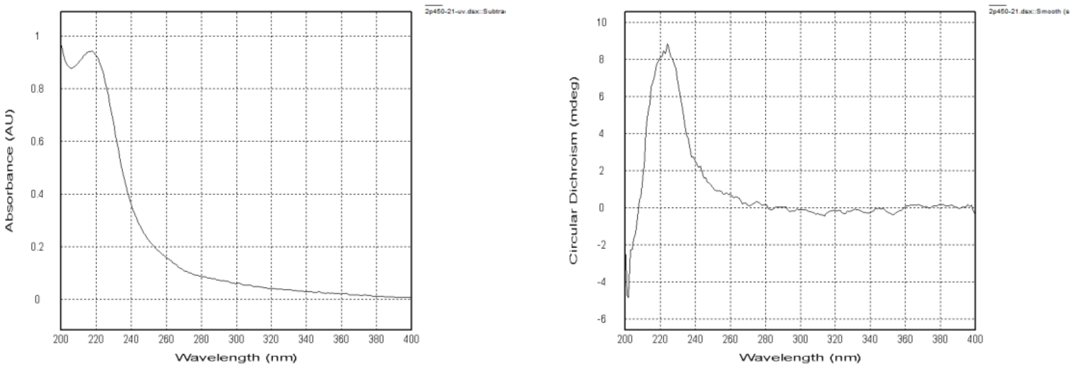

Figure S36. UV and ECD of compound **14** (CD_3OD)

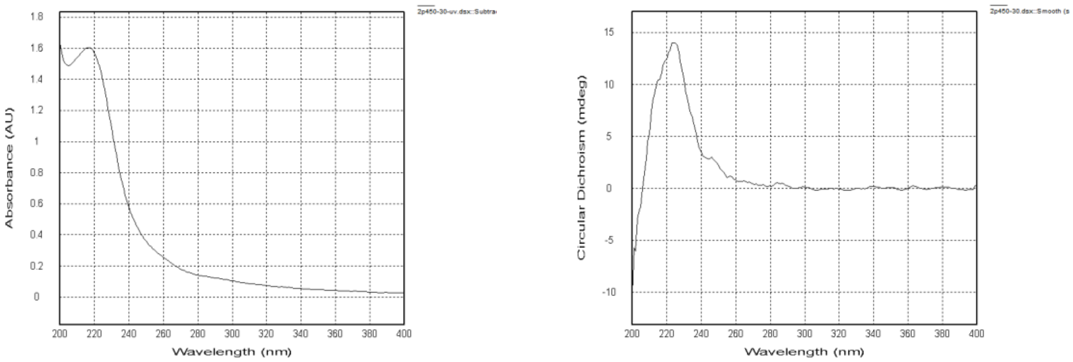

Figure S37. UV and ECD of compound **15** (CD_3OD)
